# The First Insight into the Metabolite Profiling of Grapes from Three *Vitis vinifera* L. Cultivars of Two Controlled Appellation (DOC) Regions

**DOI:** 10.3390/ijms15034237

**Published:** 2014-03-10

**Authors:** António Teixeira, Viviana Martins, Henrique Noronha, José Eiras-Dias, Hernâni Gerós

**Affiliations:** 1 Center for the Research and Technology of Agro-Environmental and Biological Sciences (CITAB), Quinta de Prados, Vila Real 5001-801, Portugal; E-Mails: antonio.teixeira@bio.uminho.pt (A.T.); vmartins@bio.uminho.pt (V.M.); henriquenoronha@bio.uminho.pt (H.N.); 2 Research Group in Applied Plant Biology and Innovation in Agrofood-Agrobioplant, Department of Biology, School of Sciences, University of Minho, Campus de Gualtar, Braga 4710-057, Portugal; 3 National Institute of Agrarian and Veterinary Research (INIAV), Quinta da Almoinha, Dois Portos 2565-191, Portugal; E-Mail: eiras.dias@inrb.pt

**Keywords:** Alvarinho, Arinto, Padeiro de Basto, grape metabolome, Vinho Verde, *Vitis vinifera*, principal component analysis

## Abstract

The characterization of the metabolites accumulated in the grapes of specific cultivars grown in different climates is of particular importance for viticulturists and enologists. In the present study, the metabolite profiling of grapes from the cultivars, Alvarinho, Arinto and Padeiro de Basto, of two Portuguese Controlled Denomination of Origin (DOC) regions (Vinho Verde and Lisboa) was investigated by gas chromatography-coupled time-of-flight mass spectrometry (GC-TOF-MS) and an amino acid analyzer. Primary metabolites, including sugars, organic acids and amino acids, and some secondary metabolites were identified. Tartaric and malic acids and free amino acids accumulated more in grapes from vines of the DOC region of Vinho Verde than DOC Lisboa, but a principal component analysis (PCA) plot showed that besides the DOC region, the grape cultivar also accounted for the variance in the relative abundance of metabolites. Grapes from the cultivar, Alvarinho, were particularly rich in malic acid and tartaric acids in both DOC regions, but sucrose accumulated more in the DOC region of Vinho Verde.

## 1. Introduction

Among the major factors influencing wine character and quality, grape variety is the most important, followed by climate, then landscape and soil. Viticulture and wine production are extremely environmentally sensitive. Finding the best terroir requires spatially appropriate data in all forms, which is becoming more available. Understanding climate structure differences between locations helps define cultivar suitability for planting and wine style and quality [1]. The physiology of grapevine has already suffered from significant impacts of global climate change in recent decades, causing significant alterations in the composition of the fruit and wine. Among several limiting factors that affect growth in Mediterranean-type ecosystems, water deficit, along with high solar radiation and extreme temperatures, are the most important ones. The combined effect of drought, high air temperature and high evaporative demand during the summer in areas like the Mediterranean basin limits grapevine yield and berry development and, consequently, wine quality [2,3].

The grape berry is considered a sink for primary key metabolites and relies on the use of available carbohydrate resources produced by photosynthesis to support its growth and development. At the harvest, berry size and quality mainly depend on water, sugars (glucose, fructose and sucrose) [4–6], organic acids (mainly malic and tartaric acids) [6,7], amino acids (arginine, proline, glutamic acid, among others) [8,9], phenolic compounds (anthocyanidins and tannins) [10,11] and aroma precursors [12]. The understanding of how and when specific metabolites accumulate and how the metabolism of the fruit of each cultivar responds to the environment is of both scientific and agronomic importance.

Over the last decade, important metabolome studies have been performed to characterize grapes from different *Vitis vinifera* cultivars, including Corvina, Merlot, Touriga Nacional, Alvarinho and Trincadeira [13–17], to compare grape berry composition at various developmental stages [18] or looking at differences between cultivars and growing seasons [19] or regions [16].

In the present study, we characterized the metabolite profiling of grapes from three Portuguese grapevine varieties—Alvarinho, Arinto and Padeiro de Basto—cultivated in two of the most important Portuguese ampelographic collections: one located in the Controlled Denomination of Origin (DOC) region of Vinho Verde in the northwest of Portugal, where wines are characterized by their freshness due to their natural acidity and are low in alcohol, and another in the central region in the DOC region of Lisboa, which normally produces elegant and aromatic red wines, rich in tannins and capable of ageing for some years in the bottle, and white wines with a fresh and citric character. These two regions were selected because in both germplasm collections, the training system, orientation of the rows, planting layout, vineyard age, yield, as well as climate conditions, including temperature, precipitation and relative humidity, are well parameterized. The varieties selected are the most used for the production of Vinho Verde. Alvarinho is considered one of the noblest white varieties in Portugal, which produced the first Portuguese mono-varietal wines with intense citrus and tropical flavors and fresh acidity. The classic profile of Arinto tends to be pale wines, aromatic with green tonality and excellent acidity. Padeiro de Basto wines are ruby to garnet-red color with a distinctive aroma and taste, harmonious and flavorful [17]. The unique character of Vinho Verde wines at the world level may result from the terroir and the variety chosen.

More than 200 metabolites were identified by gas chromatography-coupled time-of-flight mass spectrometry (GC-TOF-MS), 83 of which were unequivocally identified. Free amino acid content in grape tissues from the three cultivars grown in both DOC regions was also determined with an amino acid analyzer. Results showed that the grape variety and DOC region both accounted for the variance in relative abundance of the metabolites in the berry. Alvarinho grapes were rich in sucrose, in malic and tartaric acids and in amino acids, which may account for the typicity of Alvarinho wines produced in northern Portugal in the DOC region of Vinho Verde.

## 2. Results and Discussion

### 2.1. Results

**Figure 1.**
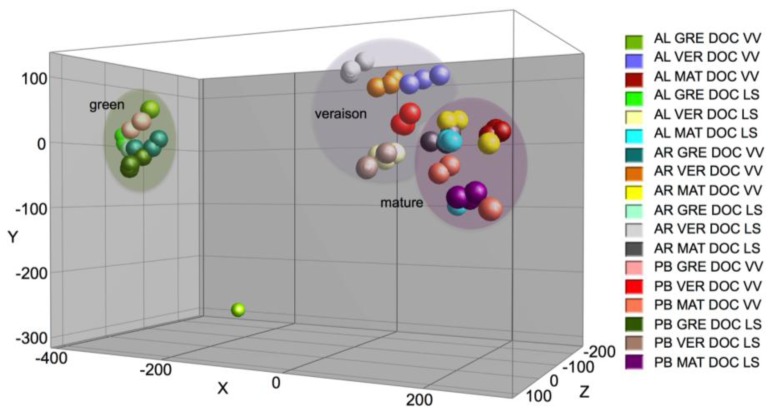
Principal component analysis (PCA) plot of Alvarinho (AL), Arinto (AR) and Padeiro de Basto (PB) metabolites in grape berries from the DOC VV (Vinho Verde) and DOC LS (Lisboa) regions performed by gas chromatography-coupled time-of-flight mass spectrometry (GC-TOF-MS). Each color represents a developmental stage (GRE, green pea; VER, veraison; MAT, mature), cultivar and region.

Grapes were harvested at green pea, veraison and mature stages (at 18 °Brix) for metabolome analysis by GC-TOF-MS. A total of 218 metabolites were detected in the grape samples, 83 of which were unequivocally identified, ranging from sugars, organic acids, amino acids and polyols (Table S1). A principal component analysis (PCA) plot readily discriminated the grape varieties, developmental stages and DOC regions. While this analysis showed that the region accounted for a 38.9% of the variance in the relative abundance of the metabolites (Figure 1), the cultivar accounted for 47.5% and 40.7% of variance in the DOC VV (Vinho Verde) and DOC LS (Lisboa), respectively (Figure S1). Important metabolites contributed to the observed dispersion of the samples between regions, including organic acids, such as malic and tartaric acids. Several metabolites present in minor levels, including sorbitol, ribitol, succinic acid and quinic acid in DOC VV, accounted for the variance between cultivars.

**Figure 2.**
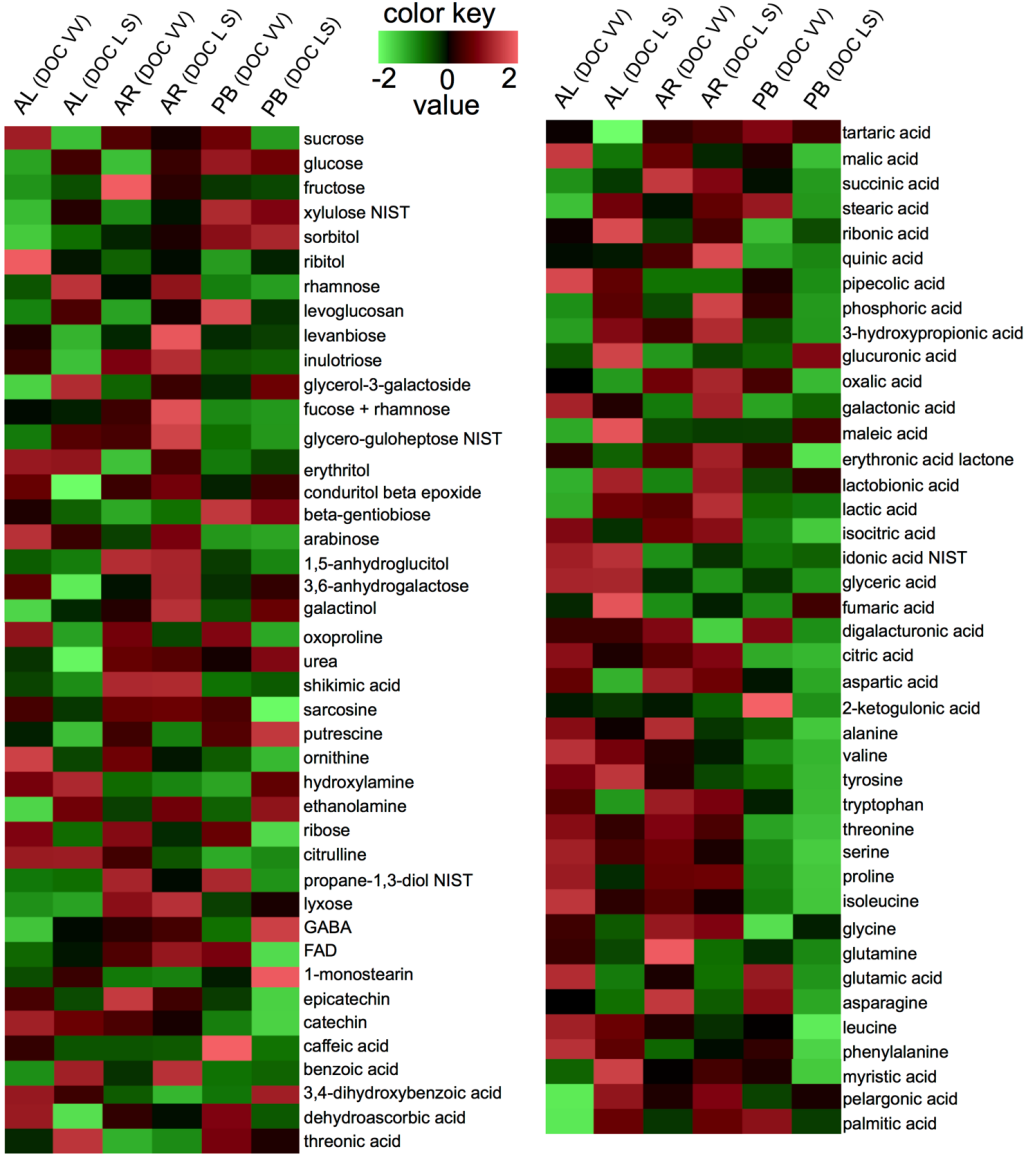
The heatmap of metabolite changes in mature grape berries from Alvarinho (AL), Arinto (AR) and Padeiro de Basto (PB) varieties cultivated in the DOC VV and DOC LS regions. Values were centered and scaled in the row direction to form virtual colors as presented in the color key.

In Figure 2, the heatmap of metabolite changes in mature grapes associated with the DOC regions is shown. For most metabolites, the trend of variation between growing regions was similar for the three selected cultivars. For instance, sucrose and malic acid decreased in all varieties from DOC VV to the DOC LS.

**Figure 3.**
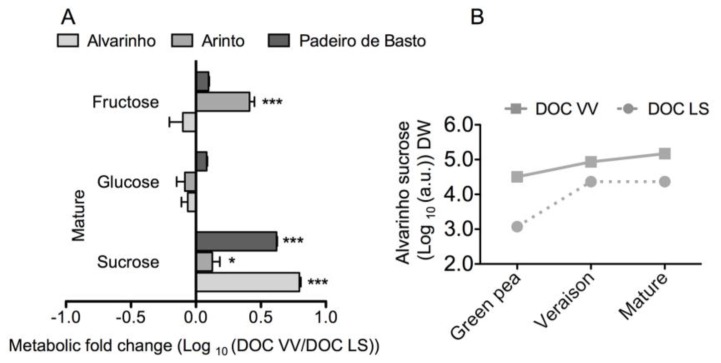
Key sugars in grape berries from Alvarinho, Arinto and Padeiro de Basto cultivated in the DOC VV and DOC LS regions. Levels of sucrose, fructose and glucose at the mature stage are the logarithmic transformed fold change (DOC VV/DOC LS) of berries from Alvarinho, Arinto and Padeiro de Basto (**A**); Logarithmic sucrose levels of Alvarinho during grape berry development and ripening (**B**). Columns represent the means ± SE (*n* = 3); asterisks denote the significance levels as comparing DOC VV to DOC LS: * *p* ≤ 0.05; *** *p* ≤ 0.001.

In general, the pattern of sugar accumulation during ripening did not change between cultivars, from veraison to mature stage. Furthermore, glucose and fructose levels did not show significant variation between regions and cultivars, as mature grapes were sampled at the same °Brix. The most noticeable difference was observed in the levels of sucrose from green pea till mature stage, which were higher in vines of the DOC VV region than of the DOC LS region (Figure 3A,B).

**Figure 4.**
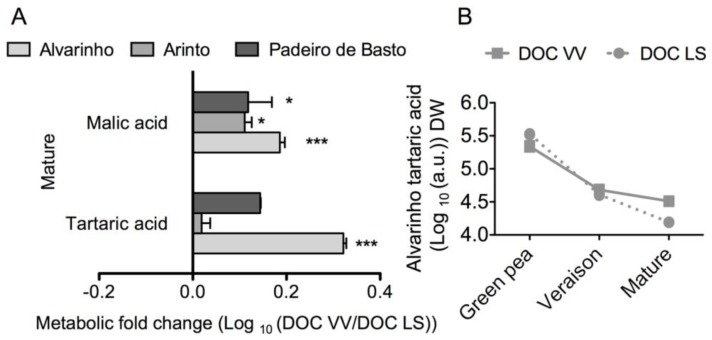
Key organic acids in grape berries from Alvarinho, Arinto and Padeiro de Basto cultivated in the DOC VV and DOC LS regions. The levels of tartaric acid and malic acid at the mature stage are the logarithmic transformed fold change (DOC VV/DOC LS) of berries from Alvarinho, Arinto and Padeiro de Basto (**A**); Logarithmic tartaric acid levels of Alvarinho during grape berry development and ripening (**B**). Columns represent the means ± SE (*n* = 3); asterisks denote the significance levels comparing DOC VV to DOC LS: * *p* ≤ 0.05; *** *p* ≤ 0.001.

Regarding organic acids, the DOC region also affected the final levels of tartrate and malate in grapes (Figure 4). Statistically significant differences were observed in mature grapes from Alvarinho, where the levels of tartaric and malic acids were two-and 1.5-fold higher in the experimental vineyards of the DOC VV than DOC LS regions, respectively. The Alvarinho cultivar seemed to be particularly rich in malic acid and tartaric acids in both DOC regions (Figure 4A and Table S1). Besides tartaric and malic acids, the present metabolome analysis also detected in all samples minor amounts of other organic acids, including citric, fumaric and succinic. Noticeably, maleic acid was very abundant in all samples and in much higher levels than its *trans*-isomer, fumaric acid (Table S1).

GC-TOF-MS analysis detected fourteen amino acids in grapes from cultivars of both regions (Table S1). To complement these results, a quantitative approach for all amino acids was performed by an amino acid analyzer system, except for tryptophan, which was quantified by HPLC. The analysis was performed in mature grapes, where nineteen of the twenty free amino acids were detected and quantified. Cysteine was not detected in all samples. The total amino acid content in grapes from each cultivar was as follows (in micrograms per gram dry weight (DW) in the DOC VV and LS regions, respectively): Alvarinho, 4415 and 3056; Arinto, 3769 and 2340; and Padeiro de Basto, 3350 and 1406 (Table 1). Arginine was the most abundant amino acid in grapes from all cultivars and regions, eventually reflecting its role as a precursor of the remaining amino acids. Proline and glutamic acid were also very abundant, with 504 and 502 μg/g DW, respectively, in grape berries from Alvarinho in the DOC VV region (Table 1).

**Figure 5.**
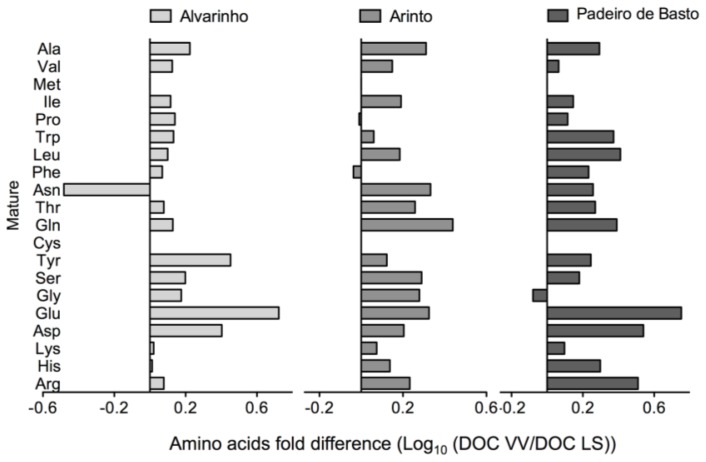
Free amino acid content in grape berries from Alvarinho, Arinto and Padeiro de Basto cultivated in the DOC VV and DOC LS regions. Values of 19 amino acid concentrations at the mature stage are the logarithmic transformed fold change (DOC VV/DOC LS) of berries from Alvarinho, Arinto and Padeiro de Basto.

Besides the observed variety-dependent differences, region conditions also affected the content of amino acids. Thus, the concentration of free amino acids is much higher in grapes from vines cultivated in the DOC VV than in the DOC LS region (Figure 5). For instance, the total amino acid content in mature grapes from Padeiro de Basto increased by 2.3-fold from the DOC LS to the DOC VV region.

**Figure 6.**
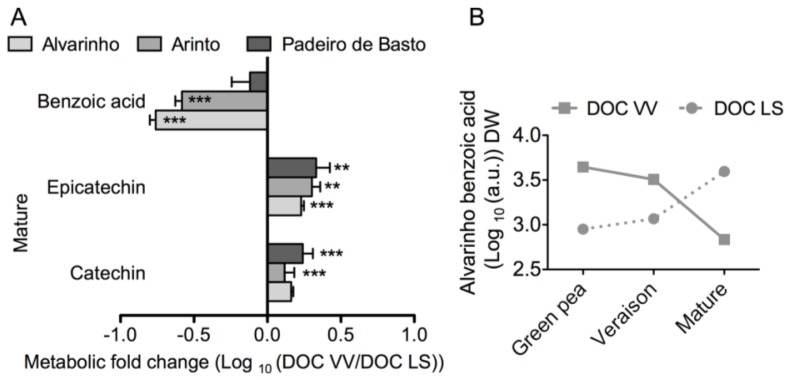
Secondary metabolites in grape berries from Alvarinho, Arinto and Padeiro de Basto from the DOC VV and DOC LS regions. The levels of benzoic acid, catechin and epicatechin at the mature stage are the logarithmic transformed fold change (DOC VV/DOC LS) of berries from Alvarinho, Arinto and Padeiro de Basto (**A**); Logarithmic benzoic acid levels of Alvarinho during grape berry development and ripening (**B**). Columns represent the means ± SE (*n* = 3); asterisks denote the significance levels comparing DOC VV to DOC LS: ** *p* ≤ 0.01; *** *p* ≤ 0.001.

Although GC-TOF-MS is more suitable for primary metabolites analysis, some secondary metabolites, including well-known hydroxycinnamates and flavan-3-ols, were also detected (Table S1). As shown in Figure 6A, the levels of benzoic acid in grapes at the mature stage from Alvarinho and Arinto cultivars were 5.7-and 3.7-fold higher in the DOC LS region than in the DOC VV region. The pattern of benzoic acid accumulation/degradation in developing grapes from Alvarinho in both regions is shown in Figure 6B. The growing region also affected the levels of catechin and epicatechin in grapes. In Alvarinho, catechins increased 1.5-fold from the DOC LS region to the DOC VV region, while epicatechins increased two-fold (Figure 6A).

**Figure 7.**
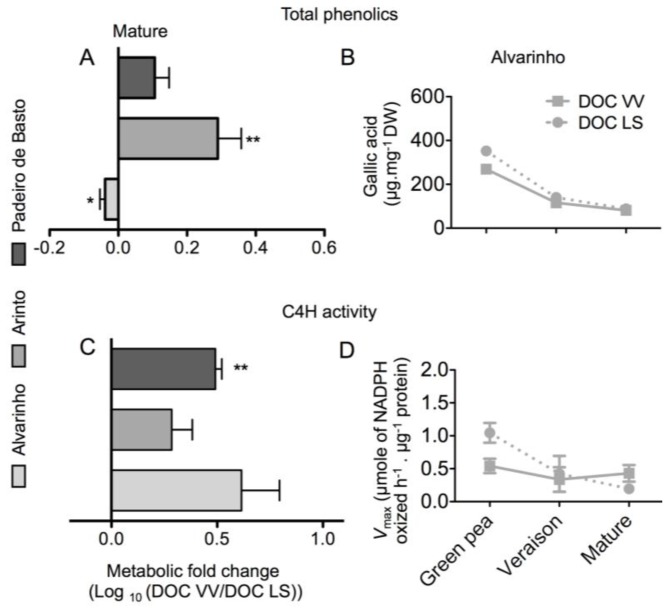
Fold difference of total phenolics and cinnamate 4-hydroxylase (C4H)-specific activity in mature grapes from Alvarinho, Arinto and Padeiro de Basto cultivated in the DOC VV and DOC LS region (**A**,**C**); Total phenolics and 4-hydroxylase (C4H)-specific activity during grape berry development in the Alvarinho variety (**B**,**D**). Values are the mean ± SE (*n* = 3); asterisks denote the significance levels comparing DOC VV to DOC LS: * *p* ≤ 0.05; ** *p* ≤ 0.01.

Total phenolics were measured by the classic Folin–Ciocalteu colorimetric method. Mature grapes from Arinto and Padeiro de Basto cultivated in the DOC VV region accumulated more phenolics than those from the same cultivars grown in the DOC LS region, by 92% and 47%, respectively (Figure 7A). However, in grapes from the Alvarinho cultivar, total phenolics decreased from the DOC VV region to the DOC LS region, although not significantly. When expressed by dry biomass, a consistent decrease in total phenolics from the green pea to mature stage was observed in all varieties and regions, as shown in Figure 7B, for the Alvarinho cultivar. Cinnamate 4-hydroxylase (C4H) catalyzes the oxidation of trans-cinnamic acid to 4-hydroxy-cinnamate, fueling the production of several secondary metabolites [20,21]. As shown in Figure 7C, the activity of C4H was higher in grapes of the DOC VV region than the DOC LS region for all cultivars, and in each cultivar, including Alvarinho (Figure 7D), a slight decrease was observed from the green pea to mature stage in both regions.

### 2.2. Discussion

Among the major factors influencing wine character and quality, the grape variety might be the most important one, followed by climate, then landscape and soil [1]. In the present study, principal component analysis (PCA) showed that the DOC region accounted for a 38.9% variance in the relative abundance of the metabolites in the grapes, and cultivar-dependent variations accounted for 47.5% and for 40.7% of the variance in the DOC VV and DOC LS region, respectively. Significant variance between shaded and exposed berries of the same cultivar were also previously observed [15], and clear differences were found between red (Trincadeira, Aragonês, Touriga Nacional) and white (Alvarinho, Arinto) grape samples [8].

Sugar concentration in the berry is dependent on developmental stage [22], environment and viticultural practices [23–25], as well as on the genotype [26–28]. In contrast, sugar concentration has been considered a relatively stable trait for a given cultivar [29]. In our study, several sugars were detected in grapes from all cultivars and sampling places, including the most important ones, glucose, fructose and sucrose, in addition to many others present in much lower levels, including rhamnose, levanbiose and inulotriose (Table 1). The amounts of glucose and fructose, which did not show significant variation between cultivars and regions, could substantially change if different criteria were used to set the maturity. However, at 18 °Brix, the observed decrease in sucrose levels in grapes of the DOC VV to the DOC LS regions was very consistent for all varieties and contributed to some extent to the observed variance between regions. This reduction in sucrose amount might be associated with the stimulation of invertase activity in the warmer region, because slightly higher levels of glucose and fructose were accumulated in the DOC LS than in DOC VV region. In agreement, high invertase activity was observed under heat stress in young tomato fruit [30]. It was shown that water deficit impact on sugar content in grapes is cultivar-dependent [31]. Thus, no significant changes were observed in Merlot sugar content under water deficit, while a significant increase in sugar content was observed in Cabernet Sauvignon berries [10,32]. Similarly, an increase in berry sugar content under water deficit was observed in Cabernet Sauvignon, but not in Chardonnay [33].

More than 20 organic acids have been identified in the grape berry, but tartaric and malic acids are by far the most abundant [34,35]. In the present study, besides the more abundant malic and tartaric acids, other organic acids, including citric acid, stearic acid and maleic acid, were also found. It has been described that organic acids in the berry are responsive to environmental conditions and viticulture practices [23,29], and important studies have been dedicated to the effects of the heat and light that reaches the berry on acid composition [15,36,37]. The observed higher amounts of malic and tartaric acids in Alvarinho and Padeiro de Basto cultivated in the DOC VV region could explain at least part of the acidic character and freshness of “vinho verde”. In this regard, organic acids contributed more to the variance between regions than between cultivars. It has been shown that elevated temperature decreases the concentration of malic acid, whereas grapevines grown in cool climates show higher amounts of malic acids [15,29,38], which is in agreement with the data of the present study. However, according to Salazar-Parra *et al.* [39], tartaric acid concentration is not significantly affected by temperature or water stress, but in Alvarinho and Padeiro de Basto, higher amounts of tartaric acid were observed in grapes of the DOC VV than of the DOC LS region. It is accepted that after veraison, tartaric acid levels in the fruit decrease, due to a dilution effect, as the volume of the fruit increases, but a strong decrease in tartaric acid per dry weight of the berry was observed here, suggesting that this acid is catabolized during ripening.

Grapevines are able to absorb both NO_3_^−^ and NH_4_^+^ ions from the soil. The reduction of NO_3_^−^ is started by nitrate reductase, forming NO_2_^−^, which is then reduced to NH_4_^+^ by nitrite reductase in the chloroplast. Besides other fates, one of the main roles of NH_4_^+^ is incorporation into amino acids. Total amino acid content is known to change between cultivars and according to microclimate conditions in response to sun exposure, but also to other causes, such as vine nutrition, vineyard management, soil type, soil moisture content, vine virus status, grape maturity and growing season [15,40–42]. GC-TOF-MS qualitative analysis (Table S1) detected only fourteen amino acids in all grape berry samples, but the quantitative approach performed with an amino acid analyzer and HPLC provided information about all twenty free amino acids in the mature grapes. In agreement with the literature [8,43–45], arginine and proline were the two most abundant amino acids in the grapes, together with glutamic acid. Proline may contribute to a sweet taste in the berry, but it can also act as an energy source, as an antioxidant and as an osmoprotectant [10,46,47]. Furthermore, total amino acid levels may have a strong impact on the secondary aromas of the wine [48]. In the present study, it was shown that Alvarinho is the cultivar with the highest content in total amino acid content in both regions, but the profile of amino acid accumulation did not change dramatically from one variety to the other. However, amino acids are consistently much less abundant in the DOC LS than in the DOC VV region, supporting that grape amino acid content is very much dependent on the terroir.

Although GC-TOF-MS and the extraction method used are more appropriate for detection of primary metabolites, some useful information on secondary metabolites was also provided by this technique. In particular, the levels of benzoic acid, catechins and epicatechin in mature grapes were found to be consistently affected by the growing region, although differences between cultivars were also noticed. In this regard, benzoic acid was much more abundant in grapes from Alvarinho and Arinto cultivated in the DOC LS than in the DOC VV region. Benzoic acid is the precursor of several common hydroxybenzoic acids, usually found in wine, such as gallic acid, gentisic acid, *p*-hydroxybenzoic acid, protocatechuic acid (3,4-dihydroxybenzoic acid), syringic acid, salicylic acid and vanillic acid [49–51]. It has been suggested that the phenolic content of grape berries is highly variable, and that each phenolic compound concentration may vary independently in response to environmental factors [15,52]. Total phenolics also seemed to follow this trend, because mature grapes from Arinto and Padeiro de Basto sampled in the DOC VV region accumulated more phenolics than those from the DOC LS region, although in Alvarinho, a small decrease from the DOC VV to the DOC LS region was found. In addition, the enzyme, cinnamate 4-hydroxylase (C4H), which mediates the production of several secondary metabolites [20,21], seemed consistently affected by the terroir, because its activity was higher in grapes from the DOC VV than from the DOC LS region.

## 3. Experimental Section

### 3.1. Characterization of the Vineyard Regions and Sample Collection

Grapes from three vine cultivars—Alvarinho, Arinto and Padeiro de Basto—were collected in the 2012 season in two different Portuguese ampelographic collections located in the Controlled Appellation (DOC) region of Vinho Verde (DOC VV) in the northwest region of Portugal (Estação Vitivinícola Amândio Galhano, −41°48′ N/8°25′ W) and in the DOC region of Lisboa (DOC LS) in the central region (Instituto Nacional de Investigação Agrária e Veterinária, −39°02′ N/9°11′ W). The soil type in the DOC VV region vineyard is Cambic Umbrisol acidic with low levels of P and K and rich in N, with low mineral colloids and high fertility at the first level [45]. Vines were trained and spur-pruned on an ascendant simple cordon system. The vineyard soil of the DOC LS region is a Calcic Fluvisol with high levels of P and K and poor in N [53]. Vines were trained and spur-pruned on an ascendant bilateral cordon system. Both vineyards were under a non-irrigated regime. The typical yield of each cultivar is as follows (in kilograms per hectare in the DOC VV and DOC LS regions, respectively): Alvarinho, 10,210 and 6000; Arinto, 14,540 and 5000; and Padeiro de Basto, 20,000 and 14,000.

**Figure 8.**
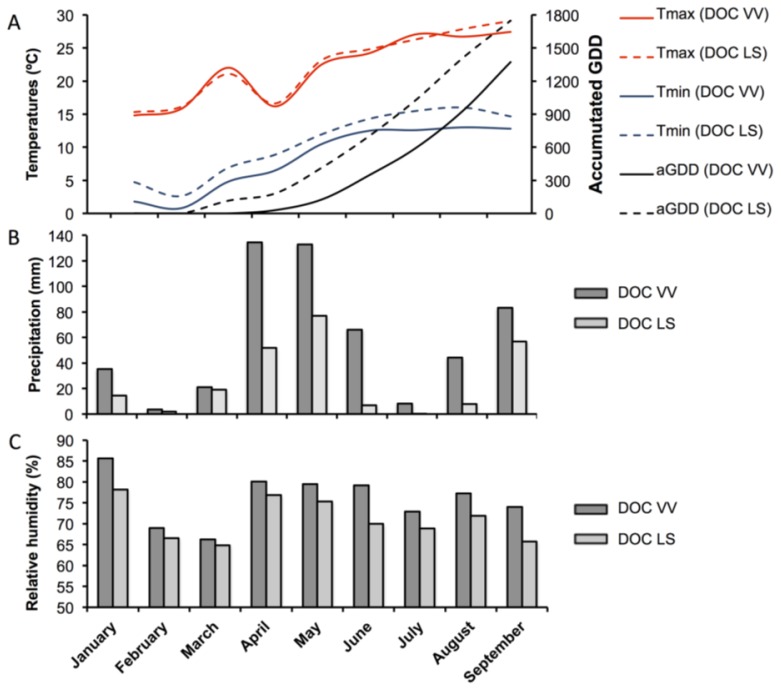
Meteorological and agrometeorological elements from the DOC VV and DOC LS regions from the January until October, 2012, season. Maximum (Tmax) and minimum (Tmin) temperatures and accumulated grown degree days (aGDD) (**A**); precipitation levels (**B**); and relative humidity (**C**).

The meteorological and agro-meteorological information collected in the 2012 season by meteorological stations located in both experimental vineyards is shown in Figure 8. The air temperature in both regions was similar, although the index of grown degree-days was higher in the southern region. In the DOC VV region, the precipitation was much higher than in the DOC LS region, and the air relative humidity was also higher.

From each variety, three clusters per vine from three different vines were collected at different phenological stages: green pea, veraison and mature at 18 °Brix. Each sample was stored separately and carried in a thermal luggage. Grapes were ground with a mortar and pestle in liquid nitrogen. The powder was stored at −80 °C for posterior use.

### 3.2. Sample Preparation for Metabolome Analysis

Grape samples were lyophilized for six days, and metabolite extraction from the lyophilized samples and analysis by GC-TOF-MS were carried out at the UC Davis Genome Center Metabolomics Laboratory, as described by Fiehn *et al*. [54]. The metabolite extraction was performed in methanol, chloroform and water in proportions of 5:2:2. After metabolite extraction and derivatization, samples were injected in split-less mode with a cold injection system (Gerstel, Mülheim an der Ruhr, Germany) and analyzed by GC (Agilent 6890, San Jose, CA, USA) using an Rtx 5Sil MS column (30 m × 0.25 mm, 0.25 μm film thickness) and an integrated guard column (Restek, Bellefonte, PA, USA). The GC was connected to a Leco Pegasus IV TOF-MS spectrometer (Leco, St. Joseph, MI, USA) controlled with Leco Chroma-TOF software v.2.32 (Leco, St. Joseph, MI, USA). Peak detection and mass spectra deconvolution were performed with Leco Chroma-TOF software v.2.25. GC-MS chromatograms were processed as described by Fiehn *et al.* [54]. Further analysis after deconvolution was made using the semi-automated workflow of the UC Davis Genome Center Metabolomics Laboratory [55]. Metabolite data were normalized using the dry weight (DW) of the lyophilized samples. Data were transformed in the logarithmic scale of base 2 (log_2_) and normalization were performed with GeneMaths XT software (Applied Maths, Sint-Martens-Latem, Belgium) was defined according to the standard deviation. Heat maps and principal component analysis (PCA) were performed to discriminate the grape berry metabolite profiles and to compare the alterations in metabolite levels throughout fruit development in different varieties and regions.

### 3.3. Quantification of Free Amino Acids

Free amino acid content was determined in lyophilized samples of mature grapes. Extraction was performed by adding 25 mL of Milli-Q H_2_O to 1 g of grape berry powder, and the quantification of natural free amino acids (excluding tryptophan) was assessed in a Biochrom 30 Amino Acid Analyzer with a weak acidic cation exchange resin acting as the stationary phase (200 × 4.6 mm column) and a number of weak acidic Li-citrate buffers acting as the mobile phase. Stepwise pH, temperature and salt concentration gradients were applied. Detection after post column derivatization with Ninhydrin (135 °C ) at 570 or 440 nm was performed (Ansynth Service B.V., Roosendaal, The Netherlands). For tryptophan quantification, the sample solutions were diluted in Milli-Q H_2_O (1:10), and analysis was performed using a Beckman System Gold HPLC (Beckman, Brea, CA, USA) equipped with an Allsphere C8 column, 250 × 4.6 mm, stationary phase, (P.J. Cobert Associates, Inc. St. Louis, MO, USA) and using a phosphate buffer/MeOH gradient (mobile phase). Detection was performed by fluorometry, with the emission wavelength set at 340 nm and the excitation wavelength set at 280 nm (Ansynth Service B.V., Roosendaal, The Netherlands).

### 3.4. Total Phenolic Content

Total phenolics were quantified by the Folin–Ciocalteu colorimetry method [56]. Total phenolics from 100 mg of grape berry powder were extracted in 1 mL of methanol (100%). The homogenates were incubated for 15 min in an orbital shaker and then centrifuged at 18,000× *g* for 20 min. Twenty microliters of supernatant were added to 1.58 mL of deionized water plus 100 μL of Folin reagent, vigorously shaken, and incubated for 5 min in the dark before adding 300 μL of 2 M sodium carbonate. After 2 h of incubation in the dark, the absorbance of the samples was measured at 765 nm. The phenolic concentrations were determined using a gallic acid (GAE) calibration curve.

### 3.5. Cinnamate 4-Hydroxylase (C4H) Activity

Extraction of total protein from frozen powder was performed as described by Stoop and Pharr [57], with some alterations. Each sample was mixed with the extraction buffer in an approximately 1:1 (*w*/*v*) powder:buffer ratio. The protein extraction buffer contained 100 mM 3-(*N*-morpholino) propanesulfonic acid (MOPS) (pH 7.5), 5 mM MgCl_2_, 1 mM EDTA, 1 mM phenylmethylsulfonyl fluoride (PMSF), 5 mM dithiothreitol (DTT), 1% (*v*/*v*) Triton X-100 and 1% (*w*/*w*) of Polyvinylpolypyrrolidone (PVPP). The homogenates were then centrifuged at 18,000× *g* for 20 min at 4 °C, and the supernatants were kept on ice. The total protein content was determined spectrophotometrically by the Bradford method [58], with bovine serum albumin (BSA) as the standard. C4H biochemical activity was assayed at 37 °C, as described by Chen *et al*. [51], with some modifications. The reaction mixture contained protein extract, 300 mM Bis-Tris propane (pH 8.9), 150 μM NADPH, 1.25 mM glucose 6-Pi and 125 μM of *trans*-cinnamic acid (different concentrations of *trans*-cinnamic acid were used for the kinetics studies) in a total volume of 1 mL. The reaction was followed at 340 nm by measuring the rate of NADPH-dependent conversion of *trans*-cinnamic acid to *p*-coumaric acid. All reactions were initiated by the addition of *trans*-cinnamic acid.

### 3.6. Statistical Analysis

The data presented consists of the results obtained from three independent experiments (samples from each grapevine being one biological replicate) for each variety at each stage and are represented as the mean ± SE. Metabolite levels of samples collected in the DOC VV region were compared with those of samples collected in the DOC LS region by the Student’s *t*-test using Prism^®^ 5 (GraphPad Software Inc., La Jolla, CA, USA) and GeneMaths XT software (Applied Maths, Sint-Martens-Latem, Belgium). In the figures and tables, the values are marked with asterisks to denote the significance levels between regions: * *p* ≤ 0.05; ** *p* ≤ 0.01; *** *p* ≤ 0.001.

## 4. Conclusions

The concept of terroir is scientifically vague and has been a topic of intense debate. While it is widely accepted that it involves geography, geology, climate, which interact with plant genetics, human factors, including variety choice, clone and rootstock, organic farming, water management, canopy management, crop load and harvest date/parameters are also important [1]. Furthermore, season-dependent transcriptional changes also account for important biochemical variability in the grape composition [59]. Why one cultivar produces a better vintage in one region than in another is so far not completely understood, nor is what particular metabolites of the grape account for the typicity of the wine. Besides the genotype and environment, other factors shown above, some of which are impossible to control in field experiments, even when vines from experimental vineyards are the targets of the study, might contribute to the chemical diversity of the grapes observed in the present study. Yet, results showed that grape variety and DOC region both affected the chemical composition of the grapes in a specific vintage. At the cultivar level, the observed region-dependent characteristics of Alvarinho grapes, including sugar, organic acid and amino acid contents, may account in some extent for the typicity of Alvarinho wine produced in northwest Portugal in the DOC region of Vinho Verde.

## Supplementary Information



## Figures and Tables

**Table 1.** Amino acid content in mature grape berries from Alvarinho, Arinto and Padeiro de Basto varieties cultivated in the DOC VV and DOC LS regions. Values are expressed as microgram per gram dry weight (DW).

Amino acids μg/g DW	Alvarinho		Arinto		Padeiro de Basto	
		
	DOC VV	DOC LS	DOC VV	DOC LS	DOC VV	DOC LS
Arg	1,237	1,034	990	579	1,047	324
His	72	70	59	43	139	70
Lys	21	20	19	16	20	16
Asp	187	74	288	180	163	47
Glu	502	95	188	89	452	80
Gly	18	12	19	10	10	12
Ser	254	161	191	98	97	64
Tyr	102	36	65	49	58	33
Cys						
Gln	172	128	319	116	258	105
Thr	328	274	321	177	80	43
Asn	30	91	43	20	76	42
Phe	136	116	68	74	53	31
Leu	93	74	49	32	67	26
Trp	122	90	147	128	132	56
Pro	504	365	394	403	300	230
Ile	64	49	45	29	21	15
Met	15	15	15	15	15	15
Val	124	93	62	44	36	31
Ala	434	259	487	238	326	166

Total	4,415	3,056	3,769	2,340	3,350	1,406
