# Supplementary material for: The First Insight into the Metabolite Profiling of Grapes from Three Vitis vinifera L. Cultivars of Two Controlled Appellation (DOC) Regions
Source: Int J Mol Sci. 2014 Mar 10;15(3):4237–54. doi: 10.3390/ijms15034237 (PMC3975394; doi:10.3390/ijms15034237)
Supplement: Supplementary file 1 [file ijms-15-04237-s001.pdf]

Supplementary Information

Figure S1. Principal component analysis (PCA) plot of Alvarinho, Arinto and Padeiro de Basto metabolites in grape berries from DOC VV (**A**) and DOC LS (**B**) regions performed by GC-TOF-MS. Each color represents a variety from the same region at green pea, veraison and mature stages.

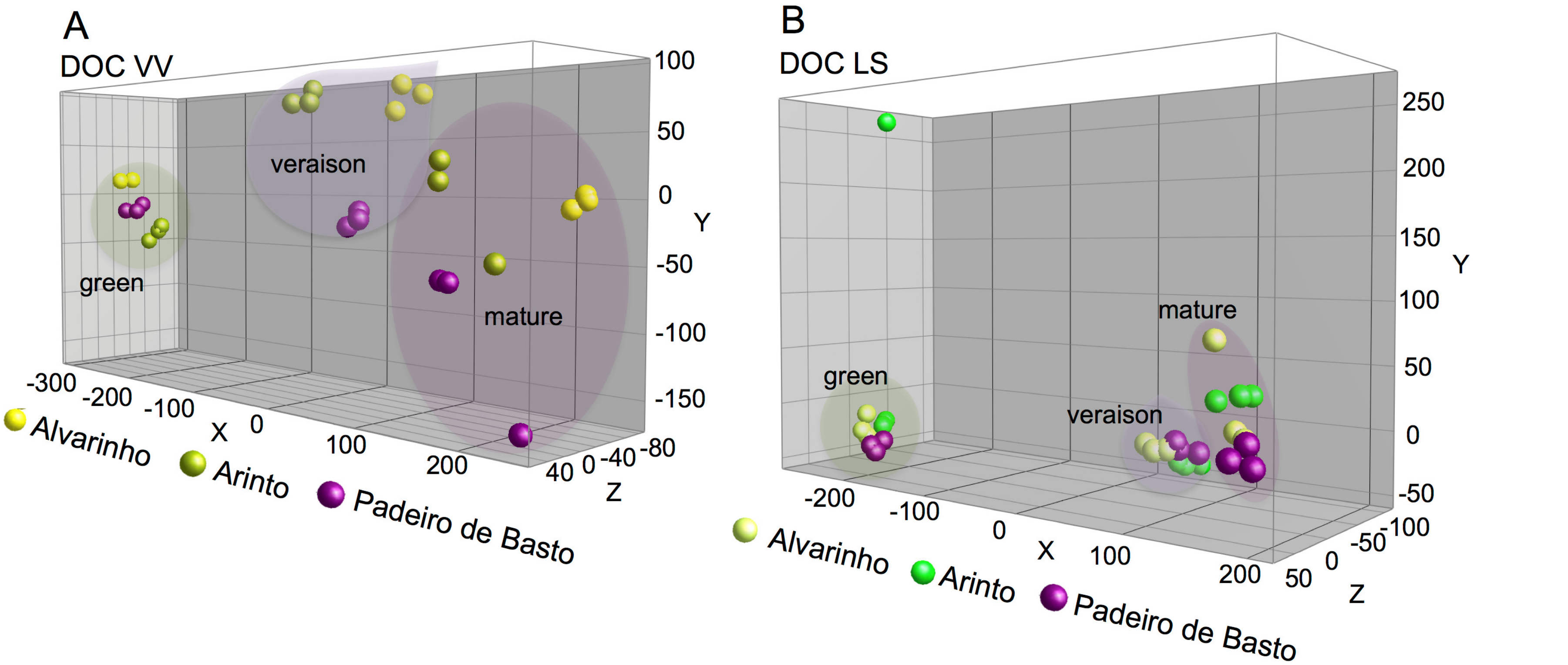

Table S1. Identified metabolites in grape berries from Alvarinho, Arinto and Padeiro de Basto varieties sampled in 2012 season in two different Portuguese ampelographic collections located in the DOC VV and DOC LS regions. Samples were collected at the green (G), veraison (V) and mature (M) stages. The data are the mean ± SD (n = 3). Asterisks denote the significance levels compared DOC VV to DOC LS: * $p \leq 0.05$; ** $p \leq 0.01$; *** $p \leq 0.001$.

No.	Metabolite	Stage	Alvarinho					Arinto					Padeiro de Basto				
			DOC VV		DOC LS		*p*	DOC VV		DOC LS		*p*	DOC VV		DOC LS		*p*
			mean	SD	mean	SD		mean	SD	mean	SD		mean	SD	mean	SD	
		G	31,913	1,587	1,192	57	***	22,028	55	1321	72	***	38,302	405	431	77	***
1	sucrose	V	84,120	2,986	23,329	261	***	56,769	1,617	16,893	525		69,028	1,895	14,928	98	***
		M	146,376	727	23,372	1,314	***	102,057	9,859	67,126	73	***	123,143	9,192	29,961	382	***
		G	99,509	1,821	88,904	578	**	105,312	28	121,881	3,764		116,848	1,457	92,160	177	***
2	glucose	V	517,015	8,247	314,704	1,173	***	484,160	3,340	378,761	4,873	***	575,225	2,324	539,029	8,520	
		M	566,069	3,132	557,481	7,239		609,915	43,094	615,787	7,921		734,059	34,156	606,181	17,545	
		G	39,513	610	44,198	132	*	56,012	293	58,680	1,448		56,300	25	44,350	71	***
3	fructose	V	1,607,560	117,535	1,296,052	4,992		1,617,848	52,406	1,258,729	46,720		583,464	16,814	1,670,545	92,695	
		M	611,684	7,250	611,345	8,516		1,628,774	407,852	623,696	25,295		739,760	50,995	591,713	29,971	
		G	541	92	1,451	354	*	399	20	984	153	***	1,975	184	3,052	186	**
4	xylulose NIST	V	155	31	179	18		157	4	171	13		263	26	278	4	
		M	143	9	179	19	**	152	22	173	183		213	54	199	42	
		G	311	35	372	13		322	30	314	257		308	26	313	8	
5	sorbitol	V	670	739	704	21		915	481	626	444		246	66	70	54	*
		M	300	54	563	148		168	21	282	111		2,206	658	2,485	165	
		G	210	36	233	51		173	35	297	67	*	132	65	166	63	
6	ribitol	V	174	33	163	2		169	16	173	12		165	29	178	15	
		M	222	37	163	20		150	74	164	167		139	38	162	27	
		G	982	75	1,073	180		861	54	1,426	420	*	1,300	7	827	109	**
7	rhamnose	V	734	209	734	48		877	35	995	60	*	901	279	883	133	
		M	642	18	1,101	490		734	117	984	112		581	163	543	51	

Table S1. *Cont.*

No.	Metabolite	Stage	Alvarinho					Arinto					Padeiro de Basto				
			DOC VV		DOC LS		*p*	DOC VV		DOC LS		*p*	DOC VV		DOC LS		*p*
			mean	SD	mean	SD		mean	SD	mean	SD		mean	SD	mean	SD	
8	levoglucosan	G	428	61	199	65		237	22	216	74	**	183	12	182	14	
		V	223	4	1,175	88		3,359	37	1,770	77		2,782	164	2,076	57	
		M	205	30	2,085	153		2,915	341	3,538	94		2,952	354	2,551	79	
9	levanbiose	G	192	21	199	65		237	22	216	74	***	183	12	182	14	
		V	2,379	143	1,175	88	***	3,229	225	1,770	77		2,782	164	2,145	128	***
		M	2,740	67	2,215	250	*	2,615	573	3,538	94		2,607	648	2,551	79	
10	inulotriose	G	320	18	186	17	**	177	27	218	39		228	29	150	13	*
		V	443	49	219	51	**	288	35	244	39		332	19	285	28	
		M	474	75	353	34		456	54	567	29		485	28	406	25	
11	glycerol-3-galactoside	G	1,130	37	1,743	42	***	799	21	1,068	874		771	56	1,127	33	**
		V	433	33	523	22	*	667	24	1,135	60	***	547	51	917	31	***
		M	217	25	770	86	***	330	54	497	91	*	396	128	591	56	
12	fucose + rhamnose	G	975	146	842	88		1,045	37	1,377	461		888	271	627	78	
		V	715	60	646	85		1,300	15	1,165	3	***	741	85	794	93	
		M	640	16	628	61		680	81	865	215		549	135	537	47	
13	glycero-guloheptose NIST	G	618	22	882	85	*	744	51	1,039	16	*	563	85	446	25	*
		V	557	46	487	13		847	40	848	76		388	60	358	38	
		M	516	22	617	37		767	83	881	54		602	61	488	80	
14	erythritol	G	177	24	379	102	*	262	24	386	79	*	274	21	278	17	
		V	558	111	362	89		577	53	806	51	**	568	77	701	87	
		M	941	45	930	146		591	140	832	46		662	104	718	65	
15	conduritol beta epoxide	G	3,223	14	2,119	82	***	3,612	78	3,754	45	**	2,762	85	1,978	37	***
		V	3,140	58	1,401	32	***	3,192	83	3,868	85		2,758	84	2,298	12	***
		M	3,020	62	1,909	91		2,986	118	3,128	51		2,589	91	2,729	65	

Table S1. *Cont.*

No.	Metabolite	Stage	Alvarinho					Arinto					Padeiro de Basto				
			DOC VV		DOC LS		*p*	DOC VV		DOC LS		*p*	DOC VV		DOC LS		*p*
			mean	SD	mean	SD		mean	SD	mean	SD		mean	SD	mean	SD	
16	beta-gentiobiose	G	359	16	322	38		295	10	354	79		352	46	308	21	
		V	837	47	326	35	***	471	31	504	209		1,557	28	698	71	***
		M	1,218	19	744	126	**	499	126	680	40		3,036	851	2,046	66	
17	arabinose	G	703	36	1,368	110		1,070	92	1,615	583		627	66	1,221	236	***
		V	309	14	267	42	**	344	58	449	18	*	298	21	331	35	
		M	275	36	254	32		242	76	264	29		231	64	228	50	
18	1,5-anhydroglucitol	G	286	79	326	47		2,260		342	70		289	32	214	24	*
		V	2,680	57	1,938	170		769	89	578	79		892	14	717	53	
		M	854	56	777	35		3,762	1,418	2,076	76		1,193	25	660	46	
19	3,6-anhydrogalactose	G	977	33	2,196	64	***	1,228	71	1,686	108		1,236	22	1,991	33	***
		V	673	21	163	19		111	5	351	274		572	49	126	21	***
		M	135	40	107	11		128	33	144	26		125	36	132	15	
20	galactinol	G	6,594	252	5,634	250	*	4,677	215	8,524	2,202	*	3,100	261	2,985	170	
		V	1139	128	994	95		1,997	43	3,506	428	**	1045	71	2,447	158	***
		M	673	101	1,051	227		1,168	213	1,716	68	*	962	216	1,379	84	
21	tartaric acid	G	218,819	9,687	339,258	713	***	325,248	4,735	335,119	41,010	*	322,475	2,717	292,950	808	**
		V	48,821	726	41,213	215	**	76,480	2,073	86,611	1,887		88,020	1,249	97,759	1,438	**
		M	32707	91	15,615	135	***	39,972	25	37,183	668		50,529	368	36,286	427	
22	malic acid	G	448,984	8,024	397,530	6,036	**	359,020	3,824	366,609	17,489		408,478	1,215	300,821	2,614	***
		V	222,137	7,145	258,947	4,217	**	304,769	7,503	518,102	11,539	***	212,593	5,111	247,296	4,075	***
		M	110,346	2,098	71,910	1,666	***	93,831	17,557	80,549	3,629		85,315	17,413	64,009	1,081	
23	succinic acid	G	1,358	1	1,047	51	**	1,205	25	1041	115		1,394	51	915	15	***
		V	370	10	135	41	**	466	70	570	54		251	55	432	18	**
		M	185	63	254	39		462	128	371	35		263	49	180	22	*

Table S1. *Cont.*

			Alvarinho					Arinto					Padeiro de Basto				
**No.**	**Metabolite**	**Stage**	**DOC VV**		**DOC LS**		***p***	**DOC VV**		**DOC LS**		***p***	**DOC VV**		**DOC LS**		***p***
			**mean**	**SD**	**mean**	**SD**		**mean**	**SD**	**mean**	**SD**		**mean**	**SD**	**mean**	**SD**	
		G	16,778	703	15,311	260		12,360	190	13,905	31		13,499	476	16,771	155	**
24	stearic acid	V	11,640	89	18,598	344	***	16,244	300	13,622	462	*	17,588	382	20,461	399	
		M	11,225	71	19,336	207	***	18,246	1,538	20,158	510		24,131	18	13,194	863	*
		G	889	40	1,269	123	*	540	44	1,211	323	**	1,529	55	1,092	40	***
25	ribonic acid	V	195	31	285	28	*	176	29	160	18		177	46	204	65	
		M	193	44	318	67	*	173	49	214	214		126	73	167	46	
		G	1,402	163	2,259	26	**	1,146	42	2,318	645	*	1,031	46	944	58	
26	quinic acid	V	329	39	473	8	**	392	28	373	18	*	187	5	213	24	
		M	220	7	216	16		257	62	389	15		153	43	165	18	
		G	525	70	633	105		159	37	134	12		200	32	206	19	
27	pipecolic acid	V	610	28	523	24	*	203	18	224	22		229	33	129	3	**
		M	681	34	402	24	***	206	39	205	13		316	35	184	19	*
		G	7,020	705	9,487	607	*	5,164	232	8,747	2,214	*	3,414	508	5,175	388	*
28	phosphoric acid	V	3,612	171	3,235	146	*	5,004	212	6,286	237	**	3,639	373	4,146	342	
		M	3,607	300	4,950	91	**	4,050	631	6,103	302	*	4,643	356	3,546	205	
		G	915	177	245	12	**	260	11	548	86		462	19	369	39	*
29	3-hydroxypropionic acid	V	446	18	159	4	***	528	66	551	40		415	22	544	50	*
		M	122	3	486	49	***	338	66	640	14	**	189	82	126	10	
		G	594	86	847	58	*	742	40	1,738	419		637	41	871	60	*
30	glucuronic acid	V	230	28	252	62		230	32	179	46		163	20	177	69	
		M	159	16	197	13	*	149	42	160	19		156	48	184	11	
		G	7,006	554	4,129	16	**	7,308	60	5,052	639		5,493	116	3,923	33	***
31	oxalic acid	V	1,827	387	635	56	*	1,256	8	1,118	100	*	1,363	288	1,968	179	*
		M	1,267	35	1,124	313		2,738	157	6,277	705		3,452	315	945	121	

Table S1. *Cont.*

No.	Metabolite	Stage	Alvarinho					Arinto					Padeiro de Basto				
			DOC VV		DOC LS		*p*	DOC VV		DOC LS		*p*	DOC VV		DOC LS		*p*
			mean	SD	mean	SD		mean	SD	mean	SD		mean	SD	mean	SD	
32	galactonic acid	G	192	54	329	8	*	220	28	394	102	*	355	12	320	8	*
		V	295	18	313	21		227	11	161	10	**	174	20	219	26	
		M	382	28	308	80		253	46	378	66		237	52	263	25	
33	maleic acid	G	90,455	3,630	57,873	2,293	**	52,111	675	62,387	4,145		72,013	2,194	60,514	1,319	**
		V	238,465	11,137	137,729	3,744	***	192,553	7,169	477,995	16,352	***	139,651	3,584	291,279	3,584	**
		M	25,731	428	93,122	2,104	***	36,034	49,966	37,627	1,076		37,489	52,499	51,244	946	
34	erythronic acid lactone	G	177	24	379	102	**	262	24	386	79		274	21	278	17	
		V	558	111	362	89		577	53	806	51		568	77	701	87	
		M	941	45	930	146		591	140	832	46		662	104	718	65	*
35	lactobionic acid	G	507	28	536	48		455	32	499	126		498	27	405	15	**
		V	483	10	323	44		203	49	381	18		204	28	193	38	
		M	123	37	382	19	**	146	13	364	23	***	181	75	151	26	
36	lactic acid	G	2,491	80	1,029	38	***	956	64	1,831	33	**	1,623	532	1,569	104	
		V	2,218	37	773	71	***	1,935	62	1,690	62	*	1,783	37	1,474	44	*
		M	637	51	1,652	29	***	1,836	151	2,266	49		944	64	786	87	
37	isocitric acid	G	1,000	32	948	36		788	33	1,125	53	*	1,057	29	849	10	***
		V	1,085	66	856	49	**	1,586	69	2,787	136	**	958	66	944	46	
		M	768	95	657	26		748	228	773	12		603	189	553	48	
38	idonic acid NIST	G	4,796	174	6,936	245	**	2,279	36	4,133	1,037	*	3,390	92	3,462	49	
		V	4,355	176	5,248	41	**	1,324	43	880	77	**	1,269	89	2,005	51	***
		M	4,475	126	5,119	216	**	1,180	255	1,884	93	*	1,350	110	1,494	90	
39	glyceric acid	G	835	69	1,259	43	**	133	4	752	69		228	190	510	13	
		V	1,371	61	1,280	47		953	120	965	57	*	927	52	599	37	***
		M	106	11	250	227		148	23	111	9		145	29	110	27	

Table S1. *Cont.*

No.	Metabolite	Stage	Alvarinho					Arinto					Padeiro de Basto				
			DOC VV		DOC LS		*p*	DOC VV		DOC LS		*p*	DOC VV		DOC LS		*p*
			mean	SD	mean	SD		mean	SD	mean	SD		mean	SD	mean	SD	
40	fumaric acid	G	1,534	73	1,381	75		1,194	109	913	78		1,067	42	954	45	*
		V	3,758	122	981	117	***	3,396	111	7924	513	***	1,569	120	4,623	222	***
		M	488	55	2,187	98	***	264	50	502	22	**	272	65	729	47	**
41	digalacturonic acid	G	265	5	286	49		265	7	510	15	*	197	18	229	31	
		V	204	24	153	23		205	51	312	24	*	158	13	178	23	
		M	169	16	169	14		177	28	144	27		177	62	151	26	
42	citric acid	G	22,943	276	18,021	226	***	18,368	65	20,286	503		23,769	297	17,391	353	***
		V	21,450	564	15,657	493	***	28,150	656	51,943	1,662	***	16,690	618	16,698	412	
		M	13,643	505	10,977	74	***	136,605	445	13,367	341		7,876	1,780	7,669	204	
43	aspartic acid	G	3,471	187	1,710	69	***	2,336	172	875	112	*	5,150	96	894	121	***
		V	1,478	45	1,723	58	**	3,098	124	3,517	143		1,691	38	1,033	70	***
		M	1,863	44	515	14	**	3,092	31	2,048	16	*	1,191	165	439	22	**
44	2-ketogulonic acid	G	11,259	104	18,726	91	***	3,948	54	7,786	64	**	5,951	69	9,033	70	***
		V	188	22	605	54	***	169	15	313	25	***	158	56	166	26	
		M	146	21	143	19		146	31	139	43		187	49	131	2	
45	oxoproline	G	17,219	5	21,098	318	***	12,582	188	6,712	194		12,335	130	7,468	118	***
		V	2,656	58	1,103	62	***	2,561	166	2,879	70	*	2,026	83	850	83	***
		M	2,481	114	994	37	***	2,249	462	1,363	360	***	2,355	624	972	50	**
46	urea	G	504	64	141	25		129	40	133	86		404	11	121	23	
		V	525	34	408	48		505	47	494	58		296	129	133	18	
		M	412	37	119	22		618	131	487	23		467	146	606	23	
47	shikimic acid	G	35,818	1329	52,362	1025	***	89,963	359	148,681	33,501	*	87,738	131	38,109	283	***
		V	2,487	6	4,440	48		1,546	106	2,314	139	***	1,782	46	3,358	92	**
		M	5,855	196	4,162	111	***	16,603	596	15,191	203		5,359	225	5,305	294	

Table S1. *Cont.*

No.	Metabolite	Stage	Alvarinho					Arinto					Padeiro de Basto				
			DOC VV		DOC LS		*p*	DOC VV		DOC LS		*p*	DOC VV		DOC LS		*p*
			mean	SD	mean	SD		mean	SD	mean	SD		mean	SD	mean	SD	
48	sarcosine	G	1,380	244	1,635	185		1,516	529	1,753	432		1,692	183	1,531	346	
		V	1,986	167	1,866	126		1,861	167	1,697	52		1,629	165	1,573	225	
		M	1,727	21	1,643	27	*	1,760	349	1,767	52		1,734	490	1,452	125	
49	putrescine	G	2,792	141	2,827	42		4,563	41	3,622	207		8,472	132	3,879	111	***
		V	829	66	533	26	**	863	24	881	34		1,112	78	963	145	
		M	754	108	447	273		916	233	557	193		979	139	1,453	70	*
50	ornithine	G	140	13	112	21		128	12	115	23		164	66	134	24	
		V	1,049	152	300	79	**	600	99	407	127		433	60	173	14	***
		M	768	83	254	123	?	479	145	303	66		232	59	149	15	
51	hydroxylamine	G	200	105	160	15		164	54	137	45		207	128	120	24	
		V	515	77	135	26	***	343	184	249	85		166	59	125	22	
		M	360	175	462	38		170	68	151	33		131	61	327	121	
52	ethanolamine	G	20,514	130	20,992	491		22,239	439	23,266	2039		17,721	346	16,600	369	*
		V	4,697	121	3,752	65	***	6,126	184	7,136	77	**	4,917	96	5,360	127	***
		M	1,644	161	4,579	224	***	2,854	480	4,591	319	*	2,545	606	5,155	167	***
53	ribose	G	170	21	279	75		113	97	352	62	*	214	28	236	21	
		V	74	38	151	6		195	17	182	19		176	12	129	60	
		M	176	20	59	8		178	13	127	82		165	31	81	38	
54	citrulline	G	186	32	167	27	***	156	13	124	18		157	20	158	20	
		V	1,996	60	516	17		957	344	685	47		714	55	361	21	***
		M	1,529	127	1,542	99		1,141	281	802	223		607	177	675	79	

Table S1. *Cont.*

No.	Metabolite	Stage	Alvarinho DOC VV mean	Alvarinho DOC VV SD	Alvarinho DOC LS mean	Alvarinho DOC LS SD	*p*	Arinto DOC VV mean	Arinto DOC VV SD	Arinto DOC LS mean	Arinto DOC LS SD	*p*	Padeiro de Basto DOC VV mean	Padeiro de Basto DOC VV SD	Padeiro de Basto DOC LS mean	Padeiro de Basto DOC LS SD	*p*
		G	16,003	1,044	10,152	461	**	12,164	477	11,314	2,525		9,025	339	5,254	480	***
55	alanine	V	22,680	855	7,600	202	***	19,565	245	15,710	363	***	6,248	106	3,258	376	***
		M	14,883	494	9,810	770	**	17,745	3,560	8,554	519	**	7,481	2,314	4,919	216	
		G	2,202	171	2,764	2	*	2,092	35	2,456	278		2,050	14	1,567	163	
56	valine	V	2,765	63	817	42	***	1,451	19	1,544	73		737	93	301	3	*
		M	3,460	42	2,449	90	***	1,982	183	1,595	105		1,134	115	801	49	
		G	555	107	697	63		600	4	719	2		556	26	560	37	
57	tyrosine	V	1,981	77	515	58	***	1,444	58	1,138	62		1,508	35	505	78	***
		M	2,737	93	737	5		2,073	156	1,325	85		1,196	21	504	93	
		G	938	128	888	38		436	17	760	9		1,550	80	680	37	***
58	tryptophan	V	2,807	107	1,557	43	***	2,903	136	2,643	74		1,913	77	1,467	108	**
		M	2,323	76	1,948	123		3,343	106	2,780	88		2,081	70	1,199	130	*
		G	2,294	286	2,300	162		1,834	18	2,611	100		1,633	144	1,875	179	
59	threonine	V	3,947	128	1,904	15	***	3,163	76	2,124	36		1,657	286	828	214	
		M	4,413	60	3,188	242	**	4,778	303	3,650	6		1,510	313	1,125	121	
		G	7,917	2	8,862	52	**	5,880	149	7,073	954		7,108	399	7,208	405	
60	serine	V	4,708	126	1,757	91	***	3,329	167	3,021	90		1,650	104	829	65	***
		M	3,335	90	2,162	4	***	2,973	303	1,920	91		1,277	70	719	59	
		G	1,434	56	1,483	57		953	59	1,400	82		997	136	1,123	163	
61	proline	V	5,467	52	1,792	4	***	2,629	67	3,179	107	**	2,368	161	1,725	36	**
		M	10,926	465	6,028	66	***	9,604	902	9,382	169		5,992	972	4,482	545	
		G	494	41	642	22		440	40	498	71		467	37	461	79	
62	isoleucine	V	994	40	497	16	***	823	59	849	34		526	87	259	30	**
		M	1,180	10	950	63		909	61	593	20		458	96	347	28	

Table S1. *Cont.*

			Alvarinho					Arinto					Padeiro de Basto				
**No.**	**Metabolite**	**Stage**	**DOC VV**		**DOC LS**		***p***	**DOC VV**		**DOC LS**		***p***	**DOC VV**		**DOC LS**		***p***
			**mean**	**SD**	**mean**	**SD**		**mean**	**SD**	**mean**	**SD**		**mean**	**SD**	**mean**	**SD**	
		G	765	8	869	50		728	42	852	77		560	9	625	53	
63	glycine	V	654	64	341	39	**	642	69	807	39	*	420	10	231	25	***
		M	466	13	459	34		718	119	600	55		368	93	496	14	
		G	23,201	561	21,806	136	*	13,598	190	6,953	589		14,111	1,243	8,558	195	**
64	glutamine	V	2,480	53	894	85	***	2,012	30	1,884	44	*	1,169	52	452	19	***
		M	793	29	600	28	**	1,633	81	483	25	**	744	42	489	78	
		G	2,542	66	2,081	76	**	1,944	84	1,149	158		2,442	21	850	9	***
65	glutamic acid	V	913	22	596	24	***	660	99	1,142	40	**	774	23	468	68	**
		M	2,506	35	405	50	***	980	47	436	91	*	2,308	42	336	20	***
		G	968	54	1,174	11		525	78	835	549		888	20	930	58	
66	asparagine	V	362	22	522	15	***	274	3	329	38		550	36	296	29	***
		M	255	24	225	89		334	81	232	58		303	61	207	7	**
		G	660	6	1,135	69	**	813	59	721	480		688	77	702	101	
67	leucine	V	1,628	7	557	46	***	959	44	1,014	15		1,819	124	113	4	**
		M	2,093	52	1,642	91	**	1,322	81	964	42		1,213	125	505	29	
		G	980	36	911	11		1,315	68	1,550	101		637	32	635	97	
68	phenylalanine	V	848	22	432	24	***	563	31	1,284	80	***	2,173	1271	866	175	
		M	1,100	16	822	95	*	524	83	658	14		812	107	371	90	*
		G	1,061	57	1,077	51		1,046	71	1,112	303		1,025	42	1,216	249	
69	propane-1,3-diol NIST	V	1,099	50	1,022	83		1,193	33	1,144	139		1,082	70	1,129	66	
		M	1,070	69	1,075	71		1,198	240	1,117	59		1,200	112	1,057	301	
		G	449	92	1,117	1305		621	78	2,554	2674		805	113	710	90	
70	lyxose	V	439	54	353	69		724	211	824	83		542	601	807	130	
		M	284	5	267	100		687	144	824	49		381	37	461	118	

Table S1. *Cont.*

No.	Metabolite	Stage	Alvarinho					Arinto					Padeiro de Basto				
			DOC VV		DOC LS		*p*	DOC VV		DOC LS		*p*	DOC VV		DOC LS		*p*
			mean	SD	mean	SD		mean	SD	mean	SD		mean	SD	mean	SD	
71	GABA	G	29,661	326	16,129	292	***	16,764	157	14,141	1,161		18,342	592	12,755	313	***
		V	13,025	626	6,981	121	***	14,101	121	19,185	402		11,416	168	8,625	387	***
		M	4,639	25	11,997	11		8,746	1492	9,419	465	***	6,117	483	14,786	103	**
72	FAD	G	1,030	19	2,556	93	***	1,389	39	2,232	556	*	1,335	41	2,324	115	
		V	125	7	159	24		119	17	199	19	**	128	27	137	26	***
		M	119	11	124	13		131	29	137	11		134	17	109	20	
73	1-monostearin	G	111	25	138	26		146	8	113	5	**	127	39	107	3	
		V	118	24	686	24	***	123	22	122	9		99	5	107	18	
		M	120	7	128	14		115	23	114	15		123	66	150	18	
74	dehydroascorbic acid	G	5,816	175	2,113	50	***	3,769	106	3,060	146		8,996	271	2,546	67	***
		V	1,727	106	686	15	***	1,821	111	503	37	***	1,904	103	837	104	***
		M	1593	24	540	39	***	1,146	216	1,025	25		1,464	345	849	104	*
75	threonic acid	G	15,350	23	24,303	340	***	19,867	216	28,681	6722		16,732	536	18,807	237	**
		V	2,487	6	4,440	48	***	1,546	106	2,314	139	**	1,782	46	3,358	92	***
		M	699	9	1,463	80	***	427	77	491	8		1,097	253	805	71	
76	myristic acid	G	295	0	335	22		323	19	285	43		307	9	347	9	**
		V	236	84	194	25		179	42	193	64		166	2	196	57	
		M	193	23	312	65	*	221	71	241	21		227	44	160	13	*
77	pelargonic acid	G	944	14	486	4	*	372	3	744	209		778	73	645	82	
		V	640	80	546	41	**	1,012	73	1,294	30		713	46	2,199	158	**
		M	297	72	1,492	43	***	528	194	1,400	59		495	103	435	27	
78	palmitic acid	G	2,687	167	3,023	74	*	2,515	226	2,511	47		2,177	51	2,656	160	**
		V	2,147	234	2,764	102	***	2,645	64	2,558	182		2,897	185	2,912	14	
		M	1,830	46	3,330	212		2,999	348	3,314	546		3,581	712	2,747	60	

Table S1. *Cont.*

No.	Metabolite	Stage	Alvarinho					Arinto					Padeiro de Basto				
			DOC VV		DOC LS		*p*	DOC VV		DOC LS		*p*	DOC VV		DOC LS		*p*
			mean	SD	mean	SD		mean	SD	mean	SD		mean	SD	mean	SD	
79	epicatechin	G	1,033	10	1,054	53		1229	60	621	128	**	1,770	55	557	19	***
		V	10,669	218	8,753	35	**	18,150	93	32,438	1,444	***	5,297	46	6,176	100	**
		M	2,296	62	1,431	66	***	4,036	549	2,208	65	**	1,667	146	674	83	**
80	catechin	G	27,779	1540	36,209	42	*	35,653	305	23,711	807		44,757	806	18,740	73	***
		V	25,011	810	22,951	23		16,303	81	30,378	838	***	4,782	59	8,388	57	***
		M	5,360	30	3,821	50	***	2,621	416	2,237	75		1,236	123	599	27	
81	caffeic acid	G	270	57	492	33	*	278	32	454	113		523	74	431	35	
		V	207	10	161	28		230	70	230	61		217	20	178	12	*
		M	175	21	152	19		152	40	152	29		239	84	145	16	
82	benzoic acid	G	4,418	77	894	68	***	753	32	3,247	303		3,436	39	2,896	14	***
		V	3,157	61	1,168	34	***	4,159	37	3,922	95		3,355	6	4,382	119	**
		M	755	2	3,942	68	***	1,406	85	4,835	23	***	985	73	927	48	
83	3,4-dihydroxybenzoic acid	G	1,244	40	1,409	78		1,812	7	1,492	77		2,327	23	1,002	22	***
		V	467	5	401	16	**	618	91	464	83		335	55	386	46	
		M	342	22	298	43		255	39	222	46		245	48	348	48	

© 2014 by the authors; licensee MDPI, Basel, Switzerland. This article is an open access article distributed under the terms and conditions of the Creative Commons Attribution license (http://creativecommons.org/licenses/by/3.0/).
